# Self-Similar Patterns from Abiotic Decarboxylation Metabolism through Chemically Oscillating Reactions: A Prebiotic Model for the Origin of Life

**DOI:** 10.3390/life13020551

**Published:** 2023-02-16

**Authors:** Dominic Papineau, Kevin Devine, Bernardo Albuquerque Nogueira

**Affiliations:** 1 London Centre for Nanotechnology, University College London, 17-19 Gordon Street, London WC1H0AH, UK; 2 Department of Earth Sciences, University College London, London WC1H0AH, UK; 3 The Centre for Planetary Sciences (CPS) at University College London/Birkbeck, London WC1H0AH, UK; 4 School of Human Sciences, London Metropolitan University, London WC1H0AH, UK; 5 Department of Chemistry, University of Coimbra, CQC-IMS, P-3004-535 Coimbra, Portugal

**Keywords:** origin of life, prebiotic chemistry, abiotic carbon, exobiology, astrobiology

## Abstract

The origin of life must have included an abiotic stage of carbon redox reactions that involved electron transport chains and the production of lifelike patterns. Chemically oscillating reactions (COR) are abiotic, spontaneous, out-of-equilibrium, and redox reactions that involve the decarboxylation of carboxylic acids with strong oxidants and strong acids to produce CO_2_ and characteristic self-similar patterns. Those patterns have circular concentricity, radial geometries, characteristic circular twins, colour gradients, cavity structures, and branching to parallel alignment. We propose that COR played a role during the prebiotic cycling of carboxylic acids, furthering the new model for geology where COR can also explain the patterns of diagenetic spheroids in sediments. The patterns of COR in Petri dishes are first considered and compared to those observed in some eukaryotic lifeforms. The molecular structures and functions of reactants in COR are then compared to key biological metabolic processes. We conclude that the newly recognised similarities in compositions and patterns warrant future research to better investigate the role of halogens in biochemistry; COR in life-forms, including in humans; and the COR-stage of prebiotic carbon cycling on other planets, such as Mars.

## 1. Introduction

The transition from the primordial lifeless Earth to a planet teeming with life occurred within a few hundred million years after the accretion of the solar system [1] and must have involved abiotic carbon cycling. This transition must also have required the adoption of primitive energy-generating chemical reactions and electron transport chains that mimicked naturally occurring abiotic reactions. While most biochemical metabolic pathways are cyclic in nature and produce energy, it remains unknown how the abiotic to biological transition took place and how these reactions evolved early to produce the patterns seen in lifeforms. What is the origin of biochemical metabolisms on the primordial Earth? How did abiotic carbon cycling give rise to complex and structured biological entities with cellular patterns? Most models of prebiotic chemistry have addressed the critical aspects of the abiotic origin of key biochemical building blocks and macromolecules from simple precursors [2,3,4], the astrophysical or terrestrial–hydrothermal origin of the simple precursors [5,6,7,8,9,10], the origin of mono- and oligomeric nucleic acids [11,12,13,14], hydrothermal vents as a likely analogue environment for prebiotic chemistry [15,16], the energetics and phosphorylation of metabolic pathways [17,18], the formation of lipid-based cellular envelopes [19,20,21], and the formation of abiotic biomorph patterns that mimic those adopted by lifeforms [22,23,24,25,26,27,28]. All these recent advances are giving us an increasingly comprehensive view of plausible abiotic pathways under early Earth conditions and how self-replicating, carbon-based, and organised structures could have emerged at the origin of life. However, one aspect that is still missing is what kind of abiotic chemistry produces the self-replicating and self-similar patterns that are seen in lifeforms, but that could also drive metabolic activity. To be relevant to the origin of life, such carbon cycling needs to happen spontaneously to generate or use out-of-equilibrium conditions such as oscillations, and to be sustained by limited reactants. These are all features fundamental to life and they partly define life as we know it. It is here argued for the first time, that on the primordial Earth, the phenomenon of abiotic carbon cycling through chemically oscillating reactions (COR) produced energy and patterns. These patterns are still expressed today in lifeforms, which leads to new scientific questions, and there are also various resemblances and analogies that can be drawn between COR and biochemistry, cellular differentiation, and molecular biology.

**Figure 1:**
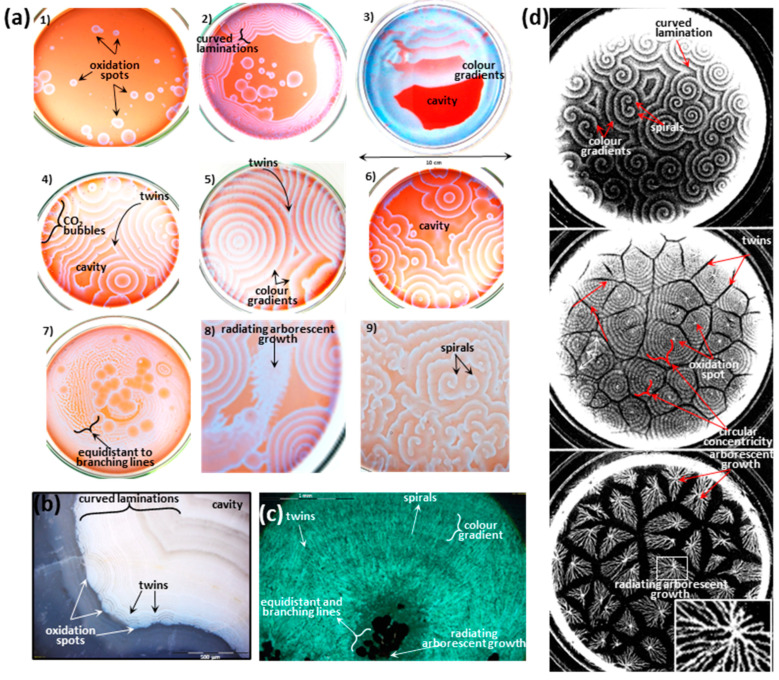
Morphological comparison of sub-millimetric to decimetric self-similar patterns in chemically oscillating experiments (left), in an agate geode and botryoidal malachite (bottom), and in growth patterns in the amoeba *D. discoideum* (right). (**a**) BZ experiments with orange-blue coloured liquid in one decimetre diameter Petri dishes. The annotations identify characteristic features of the COR self-similar patterns; (**b**) Reflected light image of a cavity in an agate geode from Mount Lyall (Gaspésie, QC, Canada) with curved laminations and perfect circularly concentric and equidistant laminations composed of kerogen gradients; (**c**) Transmitted light image of malachite botryoid from Katanga (Congo) showing equidistant circularly concentric laminations, colour gradients, radial alignment of needle-shaped crystals, twins of malachite botryoids, and a centrally located frutexites-like arborescent growth composed of kerogen; (**d**) From [42], the three dark field images, taken about 30 s apart, show cell movement during the aggregation stage (top) where *D. discoideum* migrates to form circularly concentric patterns of cyclic AMP. The onset of cell streaming (middle) is when groups of *D. discoideum* express different genes in the organised network of slime mould cells. Finally, cell stream morphology (bottom) develops into a system of different arborescent structures that are equidistant and parallel to branching lines (inset). Petri dishes in the latter experiments were 5 cm in diameter. Images from [42] are reproduced with permission; Copyright (1995) Royal Society. Other photos by D. Papineau.

The best-known COR is the Belousov–Zhabotinsky (BZ) reaction, which was discovered in the 1950s [29,30]. These reactions have, until recently, been overlooked from origin of life models, but they are relevant by being spontaneous, abiotic, out-of-equilibrium, self-catalysed, redox, and decarboxylation reactions that also form characteristic self-similar patterns (Figure 1a). COR and the BZ reaction have also been overlooked in models of mineralogy, sedimentary diagenesis, and palaeontology. For instance, there is great potential for COR to explain the patterns and substances of mineralised objects such as botryoids associated with stromatolites [31,32,33,34], concretions with fossils [1,35], granules with microfossils [36,37,38,39], and rosettes with stromatolites and microfossils [1,35,40]. These spheroidal objects all have the same characteristics, and beyond their associations with fossils, they all have circularly concentric and radially aligned mineral patterns that are attributable to COR. No other theoretical process proposed to date can satisfactorily explain these enigmatic patterns in rocks. Diagenetic spheroids have been grouped together as ‘abiotic biosignatures’, because they are elegantly explained by COR as an abiotic set of oscillating reactions represented by mineral patterns and assemblages in sedimentary rocks and, in the case of Earth, by the decarboxylation of biomass. They might also provide an explanation for the formation of agate geodes and frutexites sometimes associated with botryoidal growth (Figure 1b,c), and which also remain poorly explained by current geological models. Importantly, COR in nature do not have to involve carboxylic acids from biomass and mineralised decomposition, and instead could involve abiotically produced carboxylic acids, rendering these reactions plausible on Mars and on other hydrothermal extra-terrestrial worlds. In chemical sedimentary rocks on Earth, however, diagenetic spheroids are generally composed of carbonate, apatite, pyrite, gypsum–barite, Fe^2+^-bearing hematite, organic matter, and/or chert. When fossils are also present, these compositions confirm the mineralisation of biological remains following decomposition, and which is further consistent with the chemical composition of reactants and products in COR. These pattern-producing abiotic processes now need to be considered in the context of prebiotic chemistry.

In this contribution, COR will be discussed in light of abiotic processes closely intertwined with life and resembling a highly simplified version of metabolic cycles involving carboxylic acids. In both COR and carboxylation and decarboxylation metabolic pathways, organic acids are consumed as electron donors (‘food’) using electron acceptors, such as strong oxidants, and catalysts with redox-sensitive metal centres. Metabolic pathways and COR also involve the production of CO_2_, the generation of energy, and most notably, the production of self-similar patterns that evolve in time. Thus, the main objective of this contribution is to use detailed and specific comparisons between COR and the tricarboxylic acid (TCA) cycle, in order to explore a new model of COR for prebiotic chemistry. The approach will follow the agnostic model to understand the origin of biosignatures (i.e., the possible signatures of life), through consideration of both biological and abiotic phenomena, and specifically using objects and their patterns and substances [41]. It is likely that prebiotic reactions were influenced by thermodynamic conditions that were out-of-equilibrium such that they could proceed spontaneously and cyclically. The objects considered are lab bench experiments of COR in Petri dishes, performed under standard conditions, as well as the structure of molecules involved in both COR and metabolic pathways in lifeforms. The substances considered include reactants and products of relevant chemical reactions in COR, as well as possible metal catalysts, which will be compared to the functions and metabolism of biochemical macromolecules. Patterns will be considered using specific characteristics and geometric comparisons between the visible patterns of lifeforms seen in nature and of COR seen in lab experiments. Hence, we will establish chemical and physical similarities between abiotic COR and widespread biological metabolic pathways, such as the TCA cycle.

## 2. Observations from COR Experiments

Recipes for the production of self-similar patterns in COR include the following: in 750 cm^3^ of deionised water, add 9 g of malonic acid (propanedioic acid, C_3_H_4_O_4_), 8 g of potassium bromate (KBrO_3_), 75 cm^3^ of concentrated sulphuric acid (H_2_SO_4_), and 1.8 g of manganese sulphate (MnSO_4_·H_2_O). An alternative is to make a solution of 20 mL containing 3 mL of malonic acid solution (0.12 M), 3 mL NaBrO_3_ (0.33M), 0.416 mL H_2_SO_4_ (0.37 M), 3 mL of NaBr (60 mM), 2.4 mL of ferroin (phenanthroline ferrous sulphate, 3 mM), and 8.18 mL of dilute Tris-80. Ten to 20 mL of solution is typically used to cover the bottom of a glass Petri dish, which yields a thin film in which the patterns can be imaged due to the contrasting colours they produce. Other similar combinations of reactants that can produce patterns include iodate, ketones, diketones, citric acid, and various redox-sensitive metal catalysts including Fe, Mn, Ce, Co, Cr, and Ru (cited in [43]). However, the range of concentrations and time scales under which patterns are produced remains poorly investigated.

The following detailed description of the patterns formed by COR rests on previous terminology adopted mainly in two papers [33,34]. The most notable characteristic of the BZ patterns is that they have been described as self-similar due to the similar geometric characteristics repeated over several dimension scales, both in space and time [34]. Self-similar patterns in COR are shown in Figure 1a and include cavity shapes, twins (open-book), perfectly to imperfectly circularly concentric and equidistant laminations, columnar turbinate shapes, colour gradients in chemical waves, variable spotted, globular, zebra-striped, branching, and fingerprint-like patterns [34] (Figure 1a). The latter three pattern-types, however, tend to be formed in cavities and to oscillate at different periods. Ultimately, these three patterns can be grouped and summarised as radiating equidistant to branching lines. Other experiments carried out in the presence of colloidal silica have produced parallel banding and destructively interfered laminations, oxidation spirals, irregular to surrounded lines, open-book structures, and ripple patterns [33]. While many of these descriptive attributes are qualitative comparisons, nearly all these patterns are also reproduced by lifeforms today. Indeed, comparisons are also biochemical and geological since COR are self-catalysed by phenanthroline ferrous sulphate, and they involve compounds common in evaporitic environments.

## 3. Observations from the TCA Cycle and Lifeforms

### 3.1. Energy Production through Carboxylation and Decarboxylation

The TCA cycle is known to be governed by a series of enzymes containing a transition metal in their active sites, which is commonly known as the metallic co-factor [44]. The evolution of these enzymes on the primordial Earth was to facilitate the reactions of the TCA cycle by lowering activation energies, which resulted in the sequential carboxylation and decarboxylation reactions used today in metabolic pathways [45]. In fact, most carbon-based metabolic pathways either produce CO_2_ from the oxidation of organic molecules or the decarboxylation of carboxylic acids, or they assimilate CO_2_ through its reduction and incorporation into carboxylic acids. The reactions of the TCA cycle produce energy in the form of NADH during the formation of acetyl CoA from pyruvate, oxalosuccinate from isocitrate, succinyl Co-A from α-ketoglutarate, and oxaloacetate from malate. It also produces energy in the form of FADH_2_ when fumarate is produced from succinate and GTP when succinate is produced from succinyl-CoA. Importantly, during these sequential reactions, CO_2_ is produced from the decarboxylation steps producing pyruvate, oxalosuccinate, and α-ketoglutarate. Hence, carboxylic acids are fundamental to biochemistry and they represent molecules with stored chemical potential energy in the form of exchangeable electrons, analogous to their role in COR.

**Figure 2:**
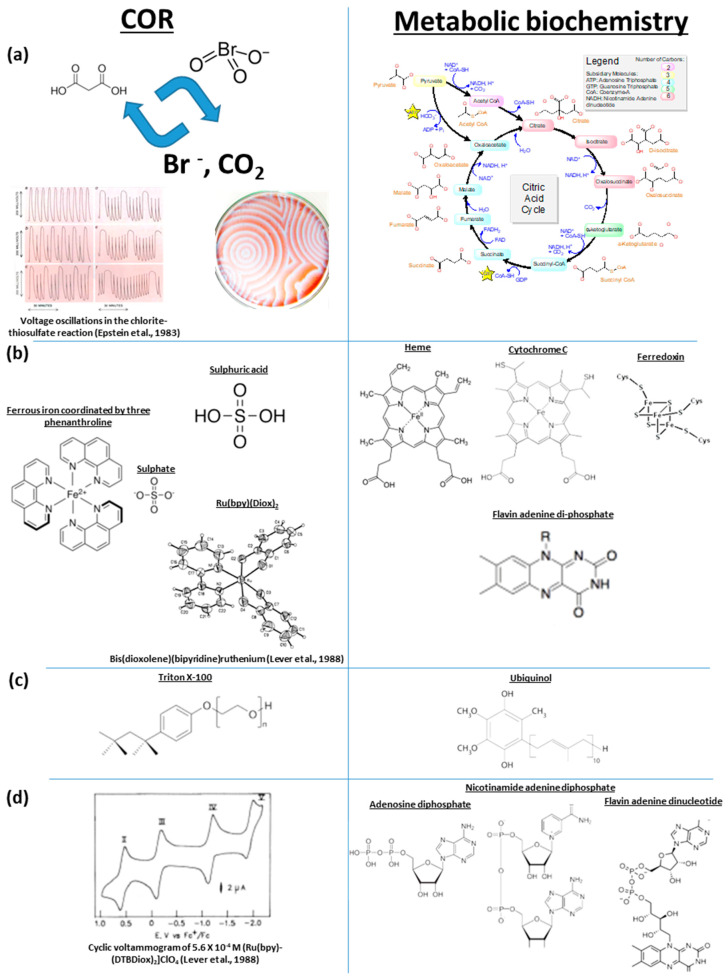
Comparison of molecular structures and chemical oscillations between metabolic biochemistry and COR. The comparisons include (**a**) carboxylic acids and cyclicity [51]; (**b**) catalysis through metal cation coordination by nitrogen in heterocyclic rings and redox-sensitive sulphur [52]; (**c**) long-chained hydrocarbons attached to functionalized benzene; (**d**) generation of chemical potential energy from the tetrabutylammonium perchlorate oscillator. Source of molecular structures: Wikipedia. Images from [51,52] are reproduced with permission; Copyright (1998) American Chemical Society and (1983) Scientific American.

COR are known to generate an electron motive force which oscillates in tandem with the diffusion of chemical waves. As this kinetic energy can be stored in chemical potential energy, future COR experiments should involve nucleotide di- and triphosphates. To proceed, metabolic reactions in the TCA cycle depend on whether acetyl-CoA is available for the reaction to continue clockwise (Figure 2). Notably, in acetyl-CoA, the acetyl is bonded to the co-factor through a thioester bond, and it is readily hydrolysed to generate a carboxylate and a thiol, and in doing so, usually forms a disulphide compound with an S-S linkage. These redox sensitive S compounds can spontaneously transfer electrons to protonated sulphur-bearing molecules. Experiments have shown that non-enzymatic reactions could have been precursors to the reverse TCA cycle and form spontaneously and efficiently in the presence of sulphate radicals [46]. In addition, the co-factor A itself is an energy-bearing nucleotide diphosphate molecule with a β-mercaptoethylamine group linked to the vitamin B5 (panthenic acid) through a bond with an amide functional group. In acidic conditions, when solutions of oxaloacetate are mixed with bicarbonate, acetyl CoA, thioacetate, and acetate, bicarbonate (a weak base) breaks down to form CO_2_ and water. This lowers the Gibbs free energy of the formation of citrate in acidic conditions more when compared to alkaline ones, even though the reactions are exergonic in both cases [47]. Hence, the spontaneous reactions during COR produce chemical potential energy through an electron motive force under a wide range of conditions, arguably from the wide environmental distribution of the TCA and reverse TCA cycles in lifeforms.

In cells, flavin reductase catalyses the reduction of flavin adenine dinucleotide (FAD) to FADH_2_, which also represents chemical potential energy. The subsequent or concomitant hydrogenation of FAD to FADH_2_ can then be involved in the production of hypochlorous acid (HClO), a halogenated intermediate with a readily exchangeable electron and proton, analogously to redox-sensitive halogens in COR. Likewise, COR are often performed with malonic acid or malonate, and both malonyl CoA and acetyl CoA play a fundamental role in the biosynthesis of fatty acid in cells. Various bacteria are able to grow aerobically or anaerobically on malonate as the sole source of carbon and energy [48]. For instance, *Malonomonas rubra* can grow anaerobically on malonate [49]. These microorganisms use decarboxylase enzymes that convert malonate into acetate and CO_2_. Decarboxylase enzymes activate malonate by forming a malonyl-thioester bond with a specific thiol-carrying cofactor of a specific acyl carrier protein (ACP) that is distinct from that operating in fatty acid synthetase. During malonate fermentation, acetate and CO_2_ are produced and the generated free energy of the decarboxylation of malonate is stored as chemical potential energy in molecules [50]. Hence, malonic acid and acetic acid have high biochemical potential energy and capacity to donate electrons through carboxyl groups and to generate chemical energy in the form of nucleoside triphosphates (such as ATP) and redox cofactors like FADH_2_. Adenosine triphosphate (ATP), in particular, is central to many metabolic pathways and forms from disequilibrium with ADP due to proton gradients in biological membranes, and is aided by ATP synthetase. Membrane electrical potentials arise both from electron transport chains inside membranes and proton gradients across membranes, which are orthogonal in spatial directions. Oxidation spots in COR radially propagate chemical waves of reduced molecules expanding as twinned circularly concentric arcs, and also have orthogonal directions. Lastly, nicotinamide adenine dinucleotide phosphate (NADH) and nucleotide triphosphates in general are widely used as energy currencies in cells: all these molecules represent chemical potential energy. However, COR do not explain all metabolic pathways, although they were likely involved in prebiotic versions of metabolic electron transfer, along with spontaneous decarboxylation reactions that produced both energy and self-similar patterns like those seen in lifeforms.

### 3.2. Patterns in Lifeforms

**Figure 3:**
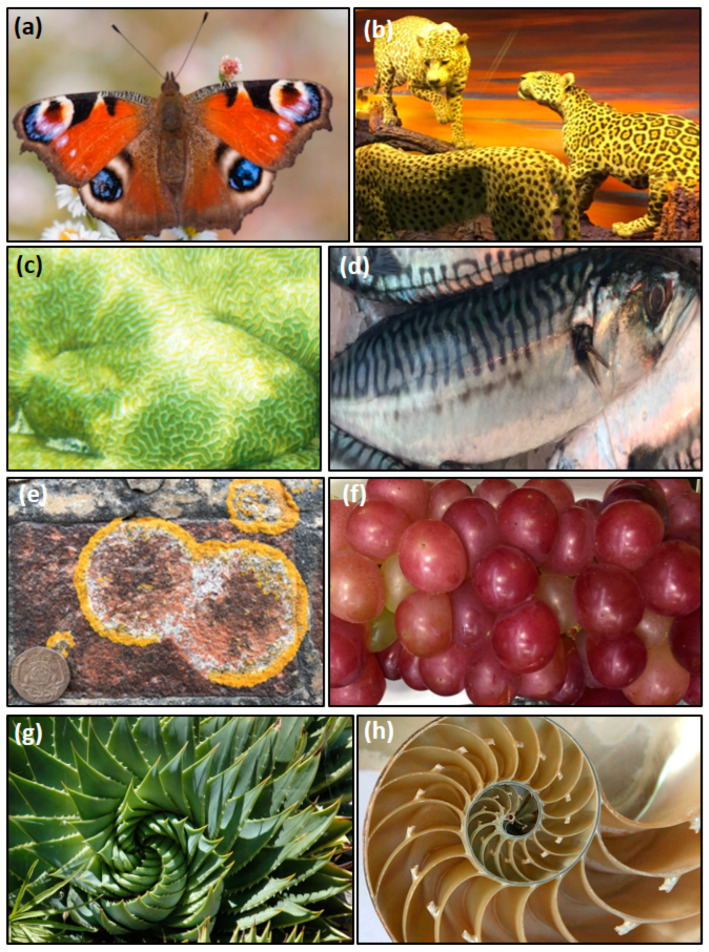
Patterns of COR in eukaryotic lifeforms. Circularly concentric spots in (**a**) butterfly and (**b**) felines. Equidistant to branching lines in (**c**) coral and (**d**) a fish. Twinned pattern in (**e**) fungus and (**f**) grapes. Spiral fractals in (**g**) cactus and (**h**) nautilus. Photos by D. Papineau.

The TCA cycle is also the ultimate biological process responsible for the production of patterns in lifeforms, producing the energy needed for cellular reproduction and differentiation, from which patterns emerge. In fact, patterns in animals, fungi, and plants arise from multicellular tissues and the variable expression of genes that triggers cellular differentiation. For instance, spots of concentric rings on butterfly wings and in the keratin that makes up the hair, feathers and scales of vertebrates, and the exoskeleton of invertebrates (Figure 3a,b) are self-similar patterns expressed due to variable cellular gene expression during growth. Other examples include pearls, which are formed in molluscs and have circularly concentric shells that constitute concretions, likely formed from the mineralisation of decaying biomass inside oysters. Similarly, spiral patterns occur in animals such as the nautilus and other molluscs (Figure 3c) and linear patterns with branching and equidistant lines are common in fish scales, corals, and animal tissue (Figure 3d–f). These patterns are all self-similar and characterised by repeating motifs; however, while they are not identical between individuals, they share similarities to COR patterns with equidistant to branching laminations (Figure 3g). Lastly, patterns of concentric rings are also seen in fungus microbe communities (Figure 3g) and even in plants (Figure 3h). All organisms on this planet are capable of expressing genes of the TCA cycle to generate energy and patterns.

Perhaps the most notable organism that makes BZ patterns is the amoeba *Dictyostelium discoideum*, which produces identical patterns as part of its lifecycle (Figure 1d; [42]). This primitive unicellular eukaryote is a slime mould with a rather unique asexual lifecycle that consists of four stages: vegetative, aggregation, migration, and culmination. During the aggregation and migration stages, *D. discoideum* produce a spiral pattern and cause amoeba migration from the central colony, following a dendritic type of pattern. This can be specifically stimulated by cyclic adenosine monophosphate (cAMP) [42]. The result is a unique growth phenomenon in eukaryotes, and in fact unique for all three domains. It has been documented that amoeba cell density forms dendritic patterns of branching and equidistant lines that radiate from the central colony, whereas the circularly concentric to spiral waves become enriched in cAMP (Figure 1d, top; [42]). This is analogous to the zebra, fingerprint-like, and dendritic patterns formed in COR with equidistant to branching laminations. In addition, when circularly concentric chemical waves become visible and meet, the same pattern as that seen in COR is produced with destructively interfered and twinned circular waves (Figure 1d, middle). In *D. discoideum*, this is the onset of cell streaming into dendritic-type branching lines bound by lines of destructive interference that form cells (Figure 1d, bottom). It is this phenomenon in particular that defines the type of multicellularity with cell-to-cell contacts established in *D. discoideum* [53], which forms groups of cells differentiated by variable gene expression [54]. These self-similar patterns are identical to those produced by purely abiotic COR and they have been mathematically modelled to quantitatively predict pattern formation during aggregation and migration stages [42]. Hence, both COR and *D. discoideum* produce patterns as circularly concentric to spiralling patterns waves, with characteristic destructively interfered chemical waves producing twins, as well as branching to parallel-aligned equidistant lines, all of which start from randomly located centres and radially diffuse over seconds to minutes timescales. Similar radiating arborescent growths occur in COR and in botryoidal malachite, but they differ with their short protrusions or bulbous terminations, respectively. Thus, equidistant to branching lines are patterns seen as ‘radiating arborescent growths’ in COR and *D. discoideum*.

### 3.3. Metal Catalysts and Molecular Structures

The compound ferroin is phenanthroline ferrous sulphate, which is a homoleptic complex that can be used to photometrically determine the concentration of ferrous iron in aqueous solutions. This is because ferrous and ferric iron are a redox switch in a longer chain of electron transfers in COR and when the iron oxidation state changes, it also changes colour. The contrast given by the colour change is what enables the visualisation of self-similar patterns in COR. Ferroin is a molecular complex that coordinates an iron cation with three phenanthroline molecules (two pyridine rings bridged by a benzene ring), which is performed by the two nitrogen atoms per phenanthroline. This six-fold coordination by phenanthroline is also found in the analogous coordination of cobalt [55] and of ruthenium [52] (Figure 2). These organometallic molecular complexes coordinate the metal cation using nitrogen atoms in heterocyclic molecules and are known to interact with deoxyribonucleic acids by involving intercalative changes in electrical potential from hydrophobic interactions with the DNA interior, or the electrical potential due to electrostatic interactions on the outer anionic coats of DNA [55]. It remains unknown whether COR can produce patterns with nucleosides or nucleotides, which could conceivably also be used as catalysts in COR and maybe also produce patterns. While Ru(bpy)(Diox) and phenanthroline cobaltous sulphate are synthetic compounds, there exist other simple analogous biochemical molecules in nature such as the tetrapyrroles cytochrome, chlorophyll (chlorin), and heme (porphyrin) (Figure 2). Tetrapyrroles all coordinate the changing oxidation state of a central iron or magnesium metal cation using nitrogen atoms inside heterocyclic rings. Cobalamin is another tetrapyrrole that plays a central catalytic role, using cobalt, in DNA synthesis, fatty acid metabolism, and amino acid metabolism. These molecules are all well known to play fundamental roles as catalytic centres for electron transfer inside the cellular membrane and are indispensable for lifeforms because they are involved in energy fixation and consumption.

Quinones, such as ubiquinol (Figure 2), are also molecules that coordinate metal cations, however they have variable repeats in a methylated alkane chain, bonded to a methylated, methoxylated, and hydroxylated benzene ring. Similarly for electron transport chains and electron bifurcation inside cellular membranes, plants and phototrophic bacteria include plastoquinone, whereas sulphate reducing bacteria use menaquinone and ferredoxin, and iron-oxidising bacteria adopted menaquinone. Quinones are analogues of the ‘Tris’ used in COR; however, they might be more related to fatty acid biosynthesis. Tris stands for tris(hydroxymethyl)aminomethane (NH_2_C(CH_2_OH)_3_), which is a widely used buffer compound in biochemistry, molecular biology, and medicine. It has a useful pH range as a buffer between 7 and 9, which is also typical of lifeforms and, while it has a distinct repeating ester bonded chain, it is also analogous to alkane and alkene chains in fatty acids. Tris plays a role in increasing the permeability of membranes and complexes with metals in aqueous solutions. However, it is unclear whether Tris undergoes reactions in COR.

Ferredoxins are relatively small redox proteins with an iron–sulphide cluster (a sub-nanoscopic Fe-S cube; Figure 2) that is coordinated by amino acids (typically cysteine or methionine), and which play a key role in electron transport chains. Ferredoxins (Figure 2) are widely used in metabolic biochemistry in all three domains of life, including in humans, and they play a role of a biological capacitor in cells, which refers to their capacity to store energy as an oscillating source of electrons. The analogy with COR and ferredoxin can thus be about the redox-sensitive catalytic power of both iron and sulphur, electron transfers, and oscillating oxidation states. It has also been argued that prebiotic chemistry involved a primitive, non-enzymatic version of metabolism, a model referred to as ‘metabolism first’ [56]. It is through this model that metals and various combinations of iron sulphide and persulphate have been recognized as essential components of a prebiotic system [46]. While COR have not yet been performed with sulphur-bearing amino acids or ferredoxins, such reactions could also conceivably produce BZ patterns, contributing to further relevance of COR for prebiotic chemistry.

Hence, tetrapyrroles, quinones, and ferredoxins are ubiquitous molecules in lifeforms with cellular functions and molecular structures, respectively, analogous to those of ferroin, Tris, and redox-sensitive sulphur used in COR. These metal cations and sulphur anions are redox centres that directly participate in electron transport chains and in energy generation. In lifeforms, energy generation is often performed through a transmembrane ATP synthetase protein, whereas in COR, an electron-motive force generates patterns through diffusion. Future studies should experiment on the reciprocal applications of COR to these natural phenomena.

### 3.4. Halogenated Compounds in COR and Lifeforms

Halogen compounds play a central role in oscillating reactions. Specifically, in the BZ reaction, the bromate anion is the key oxidizer compound [57], and the bromide ion acts as an important intermediate species, controlling the dynamics of the oscillations, including the length of the induction period [58]. Additional examples of the implication of halogen species acting as oxidants or reductants in COR include: (i) iodine, which is present in the first homogeneous isothermal chemical oscillator described, known as the Bray–Liebhafsky (BL) reaction [59]; (ii) chlorine oscillators [60], where chlorine compounds act as the oxidizer of the oscillating reaction; and (iii) reactions where both iodine and chlorine compounds are present, such as in the first systematically designed oscillating reaction, the so-called arsenite–iodate–chlorite system [61], and in the chlorate–iodine clock reactions [62]. There are also a few oscillating reactions that do not involve any halogen species, such as oscillations created by the reaction involving a sulphide ion and hydrogen peroxide [63], and the permanganate chemical oscillator in the presence of hydrogen peroxide and phosphoric acid [64]. However, the vast majority of COR require halogen compounds to occur [65], which may be attributed to the large number of oxidation states possessed by these compounds that promote the nonlinear dynamical chemistry of the COR systems. Despite the substantial number of chemically oscillatory processes that have already been reported, to our knowledge, there are no described oscillating systems where organohalogen (organic molecules functionalised with halogen elements) compounds are involved in the reaction, either as oxidants or reductants.

Naturally produced halogen compounds are fundamental to life, performing multiple biological activities, however, some of their life-related functions remain rather controversial [66]. Halogens are present in lifeforms either as inorganic compounds (e.g., halides) or as organohalogens (also known as halogenated organic compounds). Interestingly, these latter compounds are used as an energetic source by anaerobic microorganisms known as organohalide-respiring bacteria, which reduce organohalogen compounds as terminal electron acceptors in an energy-conserving respiratory process [67].

Bromine is one of the most abundant trace elements in the biosphere and has been recently considered as an essential element for life [68], though little information exists about its particular functions in lifeforms [69], apart from bromide, which is a required anion in the formation of collagen IV [68]. Recently, reactive organobromine compounds were found to be produced by human eosinophils [70,71]. More broadly, however, thousands of organobromine compounds occur in lifeforms, including in marine animals such as sponges and bryozoans, as well as in bacteria, fungi, plants, and mammals [72,73].

The same general scenario is true for iodine. Kelps, large brown algae of the Laminariales order, are the living organisms that contain the highest concentrations of iodine, having a major impact on the biogeochemical cycle of iodine [74]. In humans, iodine is an integral part of the thyroid hormones, which are organoiodine compounds with paramount biological functions in cell development, differentiation, and metabolism [75]. Additionally, non-hormonal, inorganic iodine is also present in the human body, having important biological functions in extra-thyroid tissues, such as antioxidant, apoptosis-inductor, antitumoral, and anti-atherosclerotic activities, which are, however, still poorly understood [76]. In brief, iodine-bearing thyroid hormones and other biological organic molecules are widespread in lifeforms and they play various roles in intra- and extracellular communication with cells, tissues, and organs, and in the development and physiology of many lifeforms, and probably have been since the evolution of primitive cells on the early Earth [77].

Due to the relevance of halogen compounds in COR and the importance of these elements for lifeforms, it seems essential that further investigations are designed and conducted to study the impact that different halogenated species can have on COR and on their possible role in prebiotic chemistry. In particular, natural organohalogens formed from both biological and abiotic origins can still be tested as alternative halogen reactants in COR [73]. It is foreseen that such investigations will contribute to a better understanding of bromine and iodine in biological functions, including for human health.

## 4. Abiotic COR on Mars?

Relevant environments in nature for COR are not well-known and are seldom investigated in this context. However, the required concentrations and compositions used in typical COR could conceivably be achieved in under diagenetic conditions in environments that are also evaporitic, oxygenated, iron rich, and productive. Each of these environmental conditions would favour enrichments in halogens + sulphate + salts, oxidised halogens, iron oxides, and carboxylic acids, respectively. The presumed abiotic environment on Noachian and Amazonian Mars is a good analogue for where COR could have developed in an abiotic world. Abiotic because the burden of proof for life, there and then, rests on the multitude of independent observations that will be needed to reach a broad scientific consensus. Meanwhile, recent rover missions have revealed that Martian soils contain relatively high abundances of bromide [78] and perchlorate [79,80], as well as widespread jarosite [KFe_3_(SO_4_)_2_(OH)_6_], which also contain iron with variable oxidation states. These compounds in Martian soils are consistent with evaporative conditions after their concentration by aqueous solutions on the Martian surface, which are ideal conditions for COR. Evidence for carbon cycling under these conditions includes organic molecules such as aromatic, polyaromatic, and chained alkanes functionalised with halogens, and oxygen and sulphur functional groups in Martian sedimentary rocks and soils [81,82,83]. All these compounds represent possible reactants or mineralised reaction products for COR on Mars. Hematite concretions have also been reported from sulphate-rich soils on Mars [84] and these might represent sedimentological evidence for decarboxylation reactions during COR. On Earth, abiotic diagenetic spheroids probably represent expressions of COR [34], and concretions, nodules, granules, rosettes, and botryoids are widely regarded to form during evaporitic conditions, with sulphate minerals being commonly replaced. Because these objects on Earth are commonly also associated with fossils and organic matter, they are involved in the carbon cycle. The occurrence of hematite concretions on Mars may thus indicate that prebiotic evolution of the carbon cycle on Mars reached the stage of COR. Hence, if COR are factually linked to the origin of life on Earth, the referred concretions on Mars represent important objects to investigate for the presence of possible biosignatures.

## 5. Summary

There are analogous processes, redox reactions, molecular structures, and self-similar geometrical patterns between COR and lifeforms. Analogous processes include decarboxylation of carboxylic acids, electron transport chains, reversible and cyclic reactions that end when a reactant is depleted, and energy generation through the generation of an electron motive force. Redox reactions are also analogous in both COR and metabolic pathways and they include the decarboxylation of carboxylic acids, they produce carbon dioxide and water, they use metal-bearing catalysts, and they involve sulphate, sulphuric acid, or S-bearing redox intermediates. Tetrapyrroles and quinones have functions and structures analogous to those of ferroin and Tris used in COR. Other similarities in molecular structures include phenanthroline molecules and tetrapyrroles both coordinating a redox-sensitive metal cation using four or six nitrogen atoms in molecular heterocycles. Other molecular similarities include the involvement of poly-carboxylic acids such as malonic acid as reactants (malate can drive the TCA cycle), and a possible role for a Tris analogue with quinones in electron transport chains, coordinating metal cations, and/or fatty acid synthesis. Lastly, there are similarities between COR and lifeforms with the generation of self-similar patterns that include circular concentricity, radially diffused geometry, colour gradients, randomly located reaction spots, destructively interfered chemical waves, cavity structures, parallel-aligned lines, and equidistant to branching or radiating arborescent growth. All these self-similar patterns are also observed in part of the asexual lifecycle of the model eukaryotic organism, *Dictyostelium discoideum*.

Many new experiments are needed to understand the ranges of reactant concentrations and species that can produce self-similar patterns, as well as the possible roles of bromine and iodine in biochemistry and microbiology. This new analysis of COR from the perspective of biochemistry can be used to predict that future experiments with phosphate, pyrroles, pyrimidines, nucleosides, (poly-)nucleotides, as well as with a broader range carboxylic acids and concentrations, which might also produce the same self-similar patterns. Additionally, future investigations on different metallic constituents of COR will allow a better comprehension on the correspondence of this type of chemical reaction with important biological cofactors. The broad applicability of COR to the scientific fields of prebiotic chemistry, metabolic biochemistry, molecular biology, and developmental biology resonates with their overlooked applicability to the fields of exobiology, mineralogy, sedimentary diagenesis, and palaeontology. COR are thus emblematic of abiotic and natural dissipative systems that operate in thermodynamically open and out-of-equilibrium environments, and where both energy and matter are exchanged between molecules.

With the expanding robotic exploration of the surface of Mars and of accelerating developments in prebiotic chemistry, the time is ripe for a community effort to creatively investigate COR with a broad range of conditions that could reveal further similarities between COR and the origin of fundamental processes involving molecules and cells. This type of research may eventually lead to an independent line of evidence for a hydrothermal origin of life. Lastly, if this new model is correct, it would also imply that COR now open many more avenues to understand the origin of life on Earth and beyond, as these could also explain enduring enigmas in sedimentary geology, mineralogy, biochemistry, multicellularity, developmental biology, and human health.

## Data Availability

Not applicable.
